# Brightness Discrimination in Budgerigars (*Melopsittacus undulatus*)

**DOI:** 10.1371/journal.pone.0054650

**Published:** 2013-01-18

**Authors:** Olle Lind, Sandra Karlsson, Almut Kelber

**Affiliations:** Department of Biology, Lund University, Lund, Sweden; Monash University, Australia

## Abstract

Birds have excellent spatial acuity and colour vision compared to other vertebrates while spatial contrast sensitivity is relatively poor for unknown reasons. Contrast sensitivity describes the detection of gratings of varying spatial frequency. It is unclear whether bird brightness discrimination between large uniform fields is poor as well. Here we show that budgerigars (*Melopsittacus undulatus*) need a Michelson contrast of 0.09 to discriminate between large spatially separated achromatic fields in bright light conditions. This is similar to the peak contrast sensitivity of 10.2 (0.098 Michelson contrast) for achromatic grating stimuli established in earlier studies. The brightness discrimination threshold described in Weber fractions is 0.18, which is modest compared to other vertebrates.

## Introduction

Among vertebrates, birds have relatively large eyes [1], excellent optical quality [2], [3], sophisticated colour vision [4] and high spatial acuity [5]. On the contrary, the spatial contrast sensitivity for spatial frequencies below the acuity limit is low in birds for unknown reasons [5]–[8].

A robust measure of spatial information capacity is the contrast sensitivity function (CSF), which describes sensitivity as the inverse of the contrast threshold at a range of spatial frequencies [9]. The CSF test gives an understanding of how the spatial information of repetitive patterns (such as sinusoidal gratings) is encoded by vision. However, the CSF approach ignores another fundamental category of visual stimuli that is encountered in natural scenes; large (low spatial frequency) uniform fields. This information can instead be obtained by measuring brightness discrimination thresholds.

To our knowledge, within birds, brightness discrimination has only been described in pigeons (*Columba livia*; [10]) and it is unclear how discrimination of large separate fields relates to the CSF and spatial acuity. Here, we test brightness discrimination in budgerigars (*Melopsittacus undulatus*) and compare our results to the CSF that was measured in similar light conditions [7] as well as to brightness discrimination thresholds in pigeons and other vertebrates.

## Materials and Methods

### Animals

We used two male and one female budgerigar that were fed with seed mixes supplemented with vitamins, carrots and fruit. The experiments were conducted over a time period of six weeks. On experimental days, the birds were fed in the experimental cage only, although carrots and fruit (that do not decrease the birds’ motivation in experiments) were given *ad libitum*. We did not use time constraints in the experiments and during the tests, birds could freely communicate vocally with con-specifics. The animals were kept in accordance with the ethical guidelines stated by the Swedish Board of Agriculture, (which specify appropriate housing conditions and caring routines for experimental animals). An ethical committee of the Swedish Board of Agriculture approved the experiments (permit number M190–10).

### Experimental Set Up

The experimental cage (length 1580 mm, width 860 mm, and height 670 mm) was made of matte grey metal netting except for the cage floor and one of the short ends that were made of grey plastic board. Stimuli were presented on a calibrated LCD monitor (SX3031W-H, Eizo Europe AB) behind two stimulus windows covered by Perspex boards (80×80 mm, 420 mm apart) inserted in the plastic board. Feeding boxes with removable lids were placed below each window. Birds were choosing stimuli from a starting perch, placed at the short end opposite the plastic board with the stimulus windows.

Stimuli were separated by a white opaque Perspex board that divided the cage and set the point of decision to a distance of 1268 mm (giving a visual angle of 3.6° for each stimulus and 18.3° between the stimuli). Four fluorescent tubes (Biolux L18W/965; Osram, flicker frequency 9 500 Hz) illuminated the cage giving a luminance of 8.5 cd/m^2^ reflected from the gray plastic board. The birds were monitored via a video camera placed behind the starting perch connected to a monitor visible to the observer.

### Stimuli and Procedure

As stimuli we used homogenous grey fields of varying luminance but without chromatic contrast, presented on the monitor with PowerPoint (v. 14.2.3, Microsoft Corporation). We used a two-alternative forced choice procedure. The birds were trained to visit the feeder below the brighter of two stimuli to get a food reward. A two-tone signal served to alert the bird at the onset of stimulus presentation, after which the birds were allowed to choose without time restriction. Positive choices were reinforced with access to food during 4–6 seconds. Negative choices were not punished. After each choice, the monitor turned black and the bird had to return to the starting perch to initiate a new trial.

**Figure 1:**
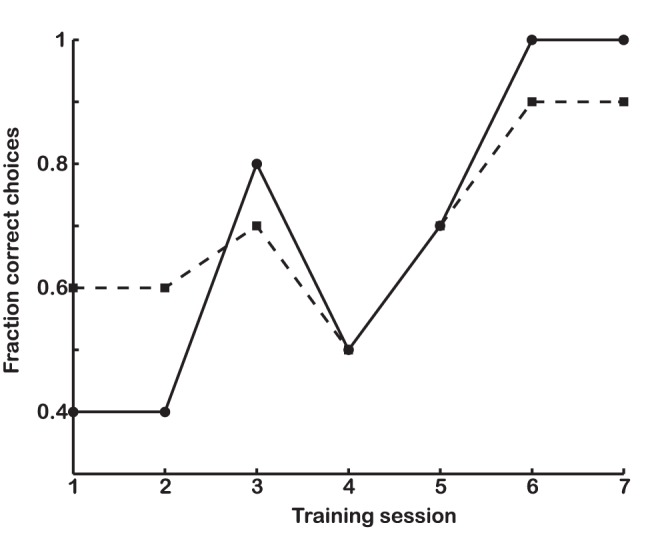
A learning curve for brightness discrimination in one budgerigar. Two stimulus pairs were used in training, one pair with a 0.38 (solid line with filled circles) and one pair with 0.18 Michelson contrast (dashed line with filled squares). In each training session, the stimulus pairs were presented 10 times each in a mixed random order. This bird qualified for testing by fulfilling the criterion of having correct choice frequencies of at least 80% for both stimulus pairs in two consecutive sessions (session 6 and 7 in this example). All birds completed training within 7 sessions.

The criteria for including the birds in test trials was an 80% correct choice frequency for stimuli with 0.18 and 0.38 Michelson contrast presented in random order during two consecutive sessions of 20 trials each (Fig. 1).

In tests, we presented the stimuli in two blocks of 16 trials. Each block comprised 4 stimulus pairs presented 4 times in random order. The blocks were designed to fully cover the threshold region while mixing stimulus pairs of high and low contrast. The stimulus pairs had Michelson contrasts of 0.34, 0.21, 0.13 and 0.04 in block 1, and 0.27, 0.17, 0.06 and 0.02 in block 2. We presented each block 10 times so that each bird made 40 choices for each stimulus pair and block two was tested after the completion of tests with block 1. The intensity of the stimulus pairs was symmetric around an average luminance of 47 cd/m^2^. Stimuli did not contain any light within the ultraviolet range of the spectrum.

We assume a binomial distribution of the data and the threshold was set to 72.5%, which corresponds to a correct choice frequency that is significantly different from random behaviour (binomial distribution, *n* = 40, p<0.01). Threshold was interpolated from a logistic function fitted to the pooled data from all three birds using a nonlinear least square procedure in the program *fit* (Matlab, v. 7.12.0.635, The MathWorks Inc.):
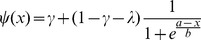
(1)where 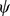 is the correct choice frequency at stimulus intensity *x*, γ is the lower asymptote of the psychometric function (fixed to 0.5), λ is the lapse rate, i.e. the difference between the upper asymptote and 1 (fixed to values between 0 and 0.2), and *a* and *b* are unrestricted fit parameters [11].

## Results

**Figure 2:**
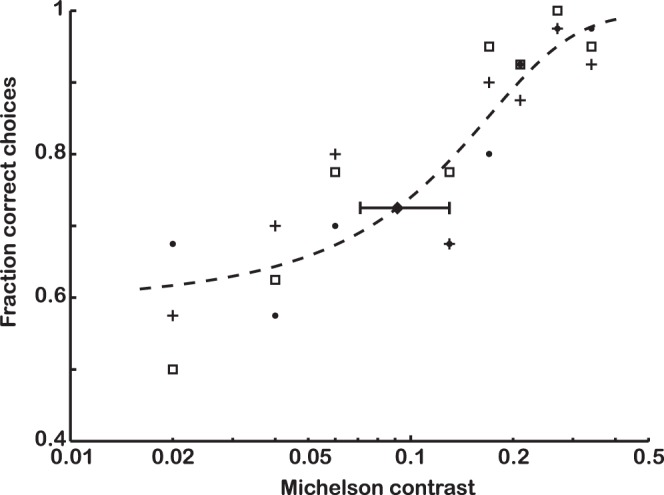
Brightness discrimination in budgerigars. Each data point represents the correct choice frequency for one bird and 40 choices. The three different markers (squares, filled circles and plus signs) indicate the results from each of the three individuals tested. A logistic function (Eq. 1) was fitted to the data (dashed line) from which the threshold was interpolated (filled diamond). Error bar shows the 68% confidence interval for the fitted function (obtained by using the program *confint* in Matlab). The brightness discrimination threshold at 72.5% correct choices (binomial test, *n* = 40, p<0.01) is 0.09 Michelson contrast units.

All birds learned the brightness discrimination task within 7 consecutive training sessions (Fig. 1). The brightness discrimination threshold derived from the pooled data of all three budgerigars is 0.09 (Michelson contrast) at 72.5% correct choices with 68% confidence limits at 0.07 and 0.13 (Fig. 2). The variation between individual birds is small (individual thresholds are 0.08, 0.11 for the males and 0.08 for the female).

## Discussion

Budgerigars need 9% Michelson contrast to discriminate between large separated achromatic fields (Fig. 2). At similar light conditions (stimulus intensity about 50 cd/m^2^), maximum contrast sensitivity to grating stimuli is 10.2, which corresponds to a Michelson contrast of 9.8% [7]. This implies that a Michelson contrast of approximately 9–10% is the general limit for achromatic discrimination in budgerigars.

In tests of brightness discrimination and spatial resolution, visual performance is at a maximum when the background illumination matches the average intensity of the stimuli [8], [12]. In this study, and earlier studies of the CSF in budgerigars [7], the background was dimmer than the stimuli. Lower contrast thresholds might be expected when background intensity is matched to the stimuli. How brightness discrimination changes with average light intensity, position and size of the test fields remains to be tested.

Brightness discrimination in budgerigars and pigeons is similar. Pigeon brightness discrimination threshold is approximately 13% Michelson contrast at the 75% correct choice frequency threshold [10] while the contrast at 75% correct choices in budgerigars is 11%. Pigeons were tested with successive stimuli [10], and it has been noted that the brightness discrimination threshold is substantially lower for stimuli presented simultaneously [12], although this has not been investigated further.

Brightness discrimination tests are very rare, and so far only two bird species (budgerigars and pigeon [10]) and a handful of mammals have been investigated (reviewed in [13]). To compare brightness discrimination in different species, we describe threshold condition with the Weber fraction, Δ*I_t/_I_s_* (where Δ*I_t_* is the intensity difference between stimuli at threshold and *I_s_* is the intensity of the standard), which is 0.18 and 0.22 in budgerigars and pigeons respectively. Compared to birds, Weber fractions are larger in horses (*Equus caballus*) and West Indian manatees (*Trichechus manatus*) (0.42–0.45 and 0.3 respectively; [14], [15]) while smaller in dogs (*Canis lupus familiaris*), harbour seals (*Phoca vitulina*), South African fur seals (*Arctocephalus pusillus*), and humans (0.11 0.14, 0.1 and 0.11 respectively; [13], [15]–[17]). There are experimental differences between these studies, yet, the general conclusion is that brightness discrimination ability in birds is in the medium range among vertebrates.

In spite of excellent optics [2], [3] and high spatial acuity [5], [8], sensitivity to stationary achromatic contrast in large fields (brightness discrimination) or repetitive patterns (CSF) such as sinusoidal gratings, is not at premium in birds. It is possible that this reflects a trade-off for some other vital visual capacity such as colour vision [6] (that is more reliable in variable light conditions [18], [19]), or a high sensitivity to moving stimuli.
